# Moderating effect of avoidance on the relationship between depression and suicidal ideation across different types of trauma exposure

**DOI:** 10.1192/bjo.2025.10953

**Published:** 2026-02-09

**Authors:** Haein Kim, Yunsu Kim, Jihye Ahn, Hyewon Yeo, Jihee Jang, Hyeri Moon, Chaeyeon Yang, Sujung Yoon, In Kyoon Lyoo, Seog Ju Kim

**Affiliations:** Department of Psychiatry, Sungkyunkwan University College of Medicine, Samsung Medical Center, Seoul, Republic of Korea; Ewha Brain Institute, Ewha Womans University, Seoul, Republic of Korea; Department of Brain and Cognitive Sciences, Ewha Womans University, Seoul, Republic of Korea; Graduate School of Pharmaceutical Sciences, Ewha Womans University, Seoul, Republic of Korea

**Keywords:** Trauma exposure, depression, PTSD symptoms, suicidal ideation, trauma type

## Abstract

**Background:**

Suicidal ideation following trauma exposure is frequently associated with depressive and post-traumatic stress disorder (PTSD) symptoms; however, the interactive effects of depression and distinct PTSD symptom clusters on suicidal ideation remain poorly understood.

**Aims:**

To examine whether specific PTSD symptom clusters – namely intrusion, avoidance and hyperarousal – moderate the association between depressive symptoms and suicidal ideation, and whether these effects vary across different trauma types.

**Method:**

Medical records of 127 psychiatric out-patients with a history of at least one traumatic event were analysed. All participants had completed the Hamilton Rating Scale for Depression, the Impact of Event Scale-Revised, and the suicidal ideation item of the Beck Depression Inventory II. Trauma types were categorised into early versus late, single versus multiple, and interpersonal versus non-interpersonal.

**Results:**

Hierarchical regression analyses identified a significant moderating effect of avoidance symptoms on the relationship between depression and suicidal ideation (*β* = 0.19, *P* = 0.012), whereas intrusion and hyperarousal symptoms did not show such effects. Specifically, higher levels of avoidance were associated with a stronger positive relationship between depression and suicidal ideation. This moderating effect was observed only among individuals with late (*β* = 0.28, *P* = 0.002), single (*β* = 0.29, *P* = 0.002) or non-interpersonal trauma (*β* = 0.34, *P* = 0.018); it was not evident among those with early, multiple or interpersonal trauma.

**Conclusions:**

These findings underscore the relevance of targeting avoidance symptoms to mitigate suicidal ideation, particularly in individuals with late-onset, single-incident or non-interpersonal trauma exposure. Exposure-based therapeutic interventions may offer particular benefit for reducing suicidal ideation among trauma-exposed individuals with depressive symptoms.

Exposure to trauma has been linked to a range of psychological disorders, most notably post-traumatic stress disorder (PTSD) and major depressive disorder.^1^ Suicidal ideation is another significant consequence of trauma exposure, although it may also be related to other non-traumatic environmental and physiological factors.^1,2^ Increased suicidal risk has been reported among trauma-exposed individuals across various populations, including military personnel, clinical cohorts and the general public.^3–5^ Given that suicidal ideation is a well-established predictor of future suicidal behaviour,^6^ its investigation in the context of trauma is of critical importance.

Accumulating evidence indicates that suicidal ideation in trauma-exposed individuals is associated with both depressive and PTSD symptoms. Depression has been found to predict suicidal ideation among veterans with a history of sexual trauma and civilians diagnosed with PTSD.^7,8^ Furthermore, depressive symptoms have been shown to mediate the relationship between childhood trauma and suicidal ideation.^9^ PTSD symptoms have similarly been associated with suicidal ideation among individuals exposed to a range of traumatic events, including life-threatening illnesses or accidents, physical assault and the unexpected loss of close others.^10,11^

PTSD and depression often co-occur and exert reciprocal influences over time.^12^ PTSD symptoms following trauma exposure have been identified as predictors of subsequent depressive symptoms.^13^ Importantly, this interplay between PTSD and depression may exacerbate suicidal ideation. Previous studies have reported that individuals with comorbid PTSD and depression exhibit significantly higher levels of suicidal ideation than those with either condition alone.^14,15^ According to the granular interaction thinking theory, an information-processing theory of the mind, mental health issues reflect maladaptive interactions between the mind and the infosphere, which are characterised by high entropy (i.e. unpredictability and uncertainty).^16^ In individuals with comorbid PTSD and depression, such high entropy may manifest as depletion of emotional resources and cognitive overload. The simultaneous interaction between depressive and PTSD symptoms can shape maladaptive cognitive processing, making it more likely that the mind will reduce its entropy towards suicidal alternatives. These alternatives may be perceived as escape from the burdens created by the comorbidity of depressive and PTSD symptoms. However, few studies to date have examined the interactive effects of PTSD and depressive symptoms on suicidal ideation in trauma-exposed populations.

Trauma type may further influence clinical outcomes. Individuals exposed to early-life trauma have demonstrated greater vulnerability to major depressive disorders, substance use disorders and specific anxiety disorders, in comparison with those exposed to trauma in adulthood.^17,18^ Multiple trauma exposures have been associated with more severe depressive and PTSD symptoms relative to single-event trauma,^19,20^ and cumulative trauma burden has similarly been linked to increased PTSD symptom severity.^4,21^ Interpersonal trauma – including physical or sexual abuse and assault – has been associated with more pronounced depressive and PTSD symptomatology than non-interpersonal trauma such as natural disasters or accidents.^22,23^ Moreover, symptom features such as restricted affect, emotional detachment, and avoidance of trauma-related thoughts or feelings are more prevalent following interpersonal trauma.^24^ These distinctions suggest that the interaction between depressive and PTSD symptoms in relation to suicidal ideation may vary depending on trauma type.

In this study, we aimed to examine whether PTSD symptoms moderate the relationship between depression and suicidal ideation across different trauma types, including early versus late trauma, multiple versus single trauma, and interpersonal versus non-interpersonal trauma. In light of evidence suggesting that PTSD comprises distinct symptom clusters – namely intrusion, avoidance and hyperarousal^25^ – we further explored the moderating effects of each of these symptom clusters to identify which dimensions most strongly influence the association between depressive symptoms and suicidal ideation. We hypothesised that PTSD symptoms would moderate the relationship between depression and suicidal ideation, and that this moderating effect would differ by trauma type.

## Method

### Participants

We reviewed medical records of out-patients who visited the Department of Psychiatry at Samsung Medical Center in Seoul, South Korea, between 2021 and 2024. From among these individuals, 127 patients with records documenting lifetime traumatic experiences and containing clinical rating scales assessing depression, PTSD symptoms and suicidality were selected for inclusion in the study. Of the 127 participants, 38 were male (29.9%) and 89 were female (70.1%). The mean age was 40.51 ± 13.93 years (range: 21–84 years). Marital status was distributed as follows: 71 participants (55.9%) were single, 49 (38.6%) were married, and seven (5.5%) were divorced or bereaved. Regarding employment status, 14 participants (11.0%) were students, 13 (10.2%) were homemakers, 61 (48.0%) were employed and 37 (29.1%) were unemployed. Detailed demographic and clinical characteristics of the participants are presented in Table 1.

**Table 1:** Demographic and clinical characteristics of the study participants (*N* = 127)

Characteristic	Mean (s.d.) or *n* (%)
Age, years	40.51 (13.93)
Gender	
Male	38 (29.9)
Female	89 (70.1)
Marital status	
Single	71 (55.9)
Married	49 (38.6)
Divorced or bereaved	7 (5.5)
Employment status^a^	
Student	14 (11.0)
Homemaker	13 (10.2)
Employed	61 (48.0)
Unemployed	37 (29.1)
Clinical symptoms	
HRSD	17.20 (6.63)
IES-R	54.45 (21.17)
IES-R intrusion	20.65 (8.72)
IES-R avoidance	17.98 (7.92)
IES-R hyperarousal	15.85 (6.92)
BDI-II suicidal ideation item	1.31 (0.90)
Trauma type	
Early	45 (35.4)
Late	82 (64.6)
Single	69 (54.3)
Multiple	58 (45.7)
Interpersonal	78 (61.4)
Non-interpersonal	49 (38.6)

HRSD, Hamilton Rating Scale for Depression; IES-R, Impact of Event Scale-Revised; BDI-II, Beck Depression Inventory II.

a. For two participants, data were not available.

The authors assert that all procedures contributing to this work comply with the ethical standards of the relevant national and institutional committees on human experimentation and with the Helsinki Declaration of 1975, as revised in 2013. All procedures involving human participants and/or patients were approved by the Institutional Review Board of Samsung Medical Center (no. 2020-11-107). Written informed consent was obtained from all participants.

### Trauma type

Traumatic experiences were identified on the basis of responses to a self-reported trauma checklist. Participants were asked whether they had experienced any of the following events over their lifetime: participation in a war or residency in a war zone; life-threatening natural disaster; serious accident; life-threatening illness; physical assault; serious crime; sexual assault; neglect or abuse by a parent or spouse; witnessing severe domestic conflict; peer bullying; or other traumatic experiences. Only traumatic events that were life-threatening, violent or seriously abusive in nature were considered for inclusion in this study.

Trauma types were classified according to three criteria: (a) age at the time of trauma exposure; (b) whether the trauma was singular or recurrent/multiple; and (c) whether the trauma was inflicted by another person. Trauma was categorised as early if it occurred before the age of 18 years and as late if it occurred thereafter. Events were designated as single trauma if they occurred only once and as multiple traumas when similar events recurred or different traumatic events were experienced. Regarding the source of trauma, events were classified as interpersonal if they were intentionally inflicted by others (e.g. physical or sexual abuse, or physical assault involving weapons) and as non-interpersonal if they involved impersonal circumstances (e.g. accidents, natural disasters, or the unexpected accidental death of a family member or close friend).

In cases in which participants reported multiple trauma types, classification was prioritised according to the presumed severity of the trauma, giving precedence to early, multiple and interpersonal trauma types, as these are commonly considered to be more severe.^23,26,27^ For instance, if a participant had experienced both physical abuse and a motor vehicle accident, this was categorised under interpersonal trauma. Of the participants, 45 (35.4%) were classified as having experienced early trauma and 82 (64.6%) as having experienced late trauma. A total of 69 participants (54.3%) reported a single traumatic event, whereas 58 (45.7%) reported multiple traumas. In addition, 78 participants (61.4%) had experienced interpersonal trauma and 49 (38.6%) had experienced non-interpersonal trauma (Table 1).

### Clinical measurements

Depressive symptoms were assessed using the Korean version of the 17-item Hamilton Rating Scale for Depression (HRSD), a clinician-administered instrument designed to evaluate depressive symptoms experienced over the preceding week.^28,29^ The total score ranges from 0 to 52, with higher scores indicating greater symptom severity. As item 3 of the HRSD evaluates suicidality, total scores excluding item 3 were also calculated and used in the analyses.

PTSD symptom severity over the past 7 days was assessed using the Korean version of the Impact of Event Scale-Revised (IES-R).^30,31^ This 22-item self-report measure uses a five-point Likert scale (0–4) to assess subjective distress caused by traumatic events. The IES-R comprises three subscales: intrusion (eight items; e.g. involuntary thoughts or feelings about the traumatic event, dissociative experiences), avoidance (eight items; e.g. efforts to evade trauma-related thoughts or cues) and hyperarousal (six items; e.g. hypervigilance, anger, exaggerated startle response). The total score ranges from 0 to 88, with higher scores reflecting greater overall distress.

Suicidal ideation was assessed using item 9 of the Beck Depression Inventory II (BDI-II), a 21-item self-report instrument that measures depressive symptom severity over the past 2 weeks.^32,33^ Each item is scored from 0 to 3, with higher scores indicating greater symptom burden. Item 9 specifically evaluates suicidal ideation, with response options ranging from 0 (‘I don’t have any thoughts of killing myself’) to 3 (‘I would kill myself if I had the chance’). Although the HRSD includes a suicidality item, this encompasses a broader spectrum of behaviours (e.g. gestures or attempts) and thus differs in scope. Given the focus of the present study on ideation rather than behaviour, item 9 of the BDI-II was selected as the primary measure of suicidal ideation.

### Statistical analysis

All statistical analyses were performed using SPSS version 29.0 for macOS (IBM Corp., Armonk, NY, USA; https://www.ibm.com/products/spss-statistics). Analysis of covariance was used to compare clinical symptom severity across trauma type groups adjusting for age and gender. When the assumption of homogeneity of variances was violated, the Quade non-parametric method was applied.^34^ Pearson correlation analyses were used to assess relationships among clinical symptom variables. Age and gender were included as covariates in all regression models. To evaluate the moderating effects of PTSD symptom severity – both overall and across the three subscales – on the relationship between depression and suicidal ideation, two sets of hierarchical regression analyses were performed. For each model, age, gender, HRSD score and IES-R score (total, or one of the subscale scores: intrusion, avoidance or hyperarousal) were entered in the first step. The interaction term between HRSD and IES-R scores was then entered in the second step to test for moderation. Further analyses stratified by trauma type group were conducted to further examine the moderating role of PTSD symptoms. When significant interaction effects were observed, simple slope analyses were carried out to clarify how the association between depression and suicidal ideation varied by level of PTSD symptoms. All continuous predictors were mean-centred before inclusion in regression models to minimise multicollinearity due to high levels of intercorrelation.

## Results

### Clinical characteristics

Males exhibited significantly higher IES-R total scores compared with females (61.29 ± 20.16 *v*. 51.53 ± 21.02; *F* = 8.15, *P* = 0.005), as well as having elevated scores on the intrusion (23.55 ± 8.51 *v*. 19.42 ± 8.56; *F* = 8.63, *P* = 0.004) and hyperarousal subscales (17.39 ± 6.40 *v*. 15.19 ± 7.07; *F* = 3.94, *P* = 0.049). No significant gender-based differences were observed for HRSD scores, the avoidance subscale of the IES-R or suicidal ideation scores. Age was inversely correlated with suicidal ideation scores (*r* = −0.30, *P* < 0.001); however, no significant associations were found between age and other clinical variables.

Significant group differences in suicidal ideation scores according to trauma type were identified, after adjustment for age and gender. Participants in the early trauma group demonstrated significantly higher suicidal ideation scores than those in the late trauma group (1.69 ± 0.90 *v*. 1.10 ± 0.84; *F* = 6.90, *P* = 0.010), and individuals with multiple trauma exposures reported significantly higher levels of suicidal ideation compared with those with a single trauma history (1.57 ± 0.92 *v*. 1.09 ± 0.84; *F* = 7.81, *P* = 0.006). Similarly, those with interpersonal trauma exhibited significantly higher suicidal ideation scores than those with non-interpersonal trauma (1.56 ± 0.91 *v*. 0.90 ± 0.74; *F* = 13.25, *P* < 0.001). By contrast, no significant differences in HRSD or IES-R scores were detected among the trauma groups.

### Correlations among depression, PTSD symptoms and suicidal ideation

HRSD scores were positively correlated with IES-R total score (*r* = 0.47, *P* < 0.001), as well as with each of its subscales: intrusion (*r* = 0.47, *P* < 0.001), avoidance (*r* = 0.33, *P* < 0.001) and hyperarousal (*r* = 0.48, *P* < 0.001). In addition, HRSD scores were significantly associated with suicidal ideation (*r* = 0.44, *P* < 0.001). Suicidal ideation scores were positively correlated with IES-R total score (*r* = 0.41, *P* < 0.001) and with all three IES-R subscales: intrusion (*r* = 0.37, *P* < 0.001), avoidance (*r* = 0.41, *P* < 0.001) and hyperarousal (*r* = 0.32, *P* < 0.001).

### Moderating effects of PTSD symptoms on the association between depression and suicidal ideation

To determine whether PTSD symptoms (IES-R) moderated the association between depression (HRSD) and suicidal ideation (BDI-Ⅱ item 9), we performed hierarchical regression analyses, controlling for age and gender. Analyses incorporating IES-R total score and the intrusion and hyperarousal subscales did not yield statistically significant moderating effects (total, *β* = 0.13, *P* = 0.105; intrusion, *β* = 0.05, *P* = 0.562; hyperarousal, *β* = 0.06, *P* = 0.489). However, as summarised in Table 2, the IES-R avoidance subscale demonstrated a statistically significant moderating effect on the relationship between HRSD scores and suicidal ideation. In the first step of the regression model, age, gender, HRSD scores and IES-R avoidance scores were included as predictors. This model was statistically significant (*R* ^2^ = 0.34, *F* = 15.42, *P* < 0.001), with age (*β* = −0.23, *P* = 0.003), HRSD (*β* = 0.33, *P* < 0.001) and IES-R avoidance (*β* = 0.29, *P* < 0.001) emerging as significant predictors. In the second step, an interaction term between HRSD and IES-R avoidance was added, resulting in a significant increase in explained variance (*R* ^2^ = 0.37, *ΔR* ^2^ = 0.03, *F* = 14.19, *P* = 0.012). The interaction term significantly predicted suicidal ideation (*β* = 0.19, *P* = 0.012), accounting for an additional 3% of the variance. Notably, this moderating effect of avoidance remained significant even when the suicide item was excluded from the HRSD score (*β* = 0.20, *P* = 0.01), whereas no significant moderating effects were observed for the other PTSD symptom clusters.

**Table 2:** Regression analysis of the moderating effect of avoidance on the relationship between depression and suicidal ideation

	Standardised coefficient *β*	*t*	*P*	*R* ^2^	*F*	*P* for *ΔF*
Step 1				0.34	15.42^***^	<0.001
Age	−0.23	−2.98	0.003			
Gender	0.06	0.74	0.460			
Depression	0.33	4.28	<0.001			
Avoidance	0.29	3.54	<0.001			
Step 2				0.37	14.19^***^	0.012
Age	−0.22	−2.89	0.005			
Gender	0.07	0.95	0.342			
Depression	0.36	4.71	<0.001			
Avoidance	0.30	3.82	<0.001			
Depression × avoidance	0.19	2.55	0.012			

*** *P* < 0.001.

**Fig. 1:**
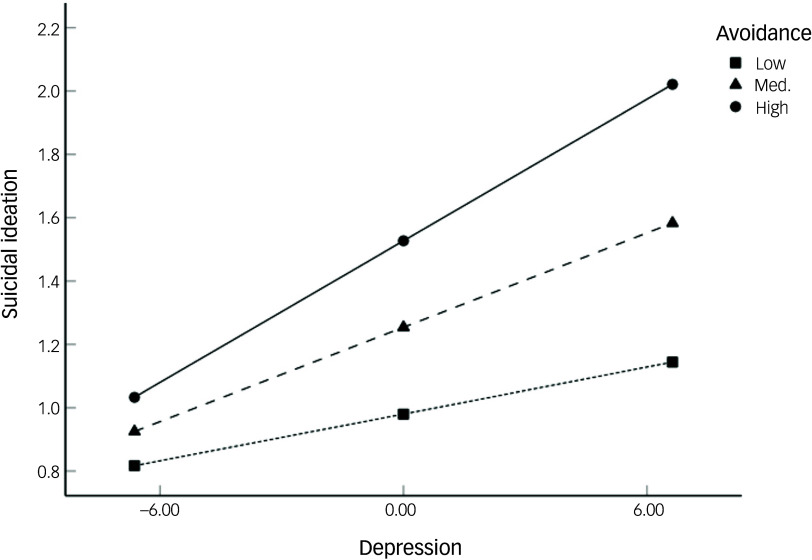
Moderating effect of avoidance on the association between depression and suicidal ideation. Med., medium.

To further elucidate this interaction, we conducted a simple slope analysis to examine the association between depression and suicidal ideation at low (−1 s.d.), medium (mean) and high (+1 s.d.) levels of avoidance (Fig. 1). Depression did not significantly predict suicidal ideation at low levels of avoidance (*B* = 0.03, *P* = 0.065), but the relationship was significant at both medium (*B* = 0.05, *P* < 0.001) and high (*B* = 0.08, *P* < 0.001) levels. These results indicate that depressive symptoms are associated with increased suicidal ideation only in the presence of moderate to high levels of trauma-related avoidance.

We further analysed the moderating effects of PTSD symptoms on the association between depression and suicidal ideation within each specific trauma type group. In the late trauma group, IES-R avoidance subscale score significantly moderated the relationship between HRSD and suicidal ideation, whereas intrusion and hyperarousal subscale scores did not yield significant moderating effects. Both steps of the hierarchical regression model were statistically significant (step 1: *R* ^2^ = 0.38, *F* = 11.68, *P* < 0.001; step 2: *R* ^2^ = 0.45, *ΔR* ^2^ = 0.07, *F* = 12.45, *P* < 0.001), and the interaction between HRSD and avoidance was a significant predictor of suicidal ideation (*β* = 0.28, *P* = 0.002). By contrast, none of the IES-R subscales demonstrated a significant moderating effect in the early trauma group.

In the single trauma group, both the initial model and the interaction model were statistically significant (step 1: *R* ^2^ = 0.41, *F* = 11.12, *P* < 0.001; step 2: *R* ^2^ = 0.49, *ΔR* ^2^ = 0.08, *F* = 12.15, *P* < 0.001), with a significant interaction effect between HRSD and IES-R avoidance (*β* = 0.29, *P* = 0.002). By contrast, no significant moderating effects of PTSD symptoms were observed in the multiple trauma group.

Among trauma type subgroups, a significant moderating effect of avoidance was also identified in the non-interpersonal trauma group. Both steps of the regression model were significant (step 1: *R* ^2^ = 0.29, *F* = 4.55, *P* = 0.004; step 2: *R* ^2^ = 0.38, *ΔR* ^2^ = 0.09, *F* = 5.26, *P* < 0.001), and avoidance significantly moderated the association between HRSD and suicidal ideation (*β* = 0.34, *P* = 0.018). By contrast, none of the PTSD symptom dimensions showed a significant moderating effect in the interpersonal trauma group.

**Fig. 2:**
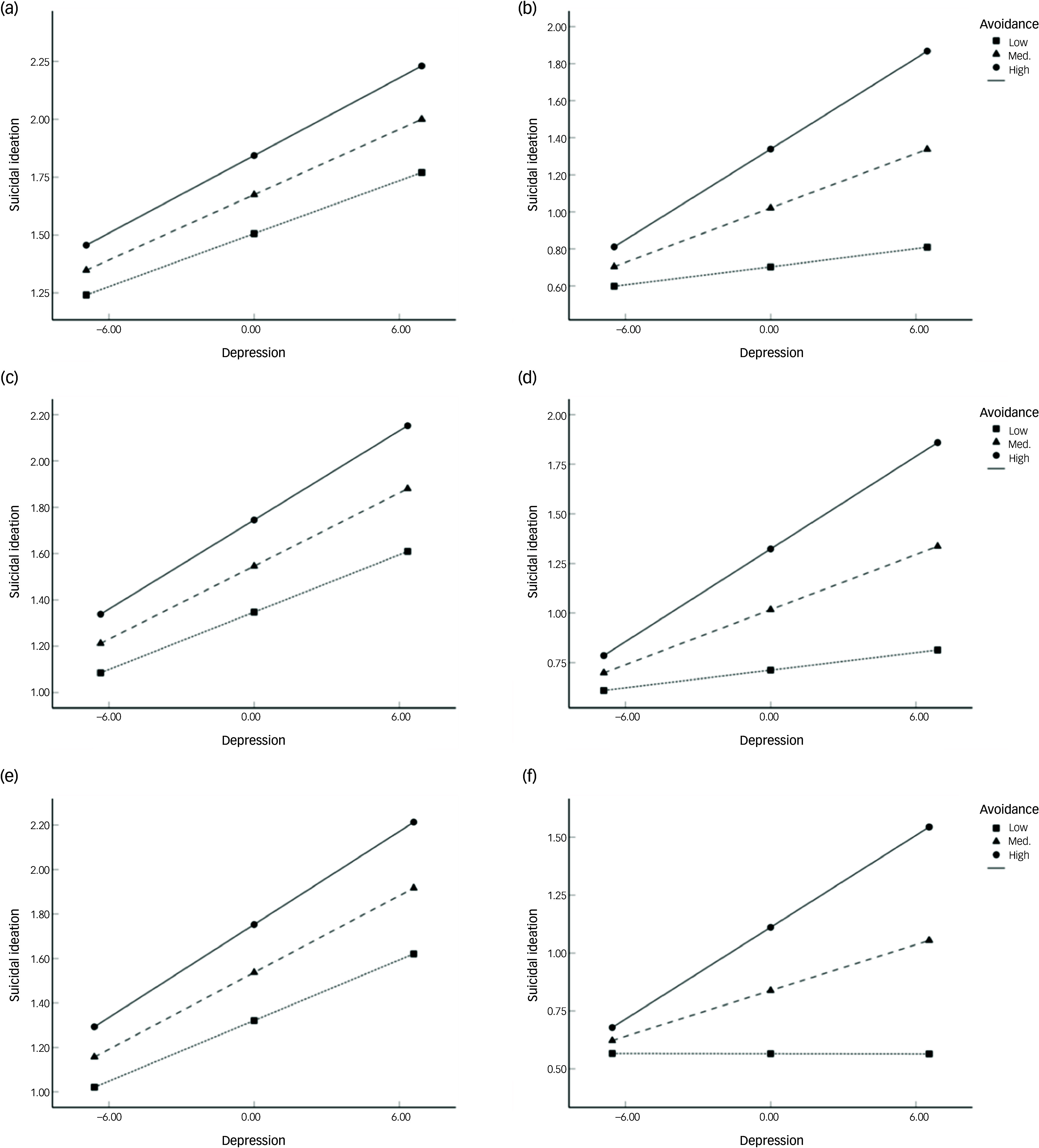
Moderating effect of avoidance on the association between depression and suicidal ideation by trauma type. (a) Early trauma group, (b) late trauma group, (c) multiple trauma group, (d) single trauma group, (e) interpersonal trauma group, (f) non-interpersonal trauma group. Med., medium.

To further interpret the observed moderating effects of avoidance, we conducted simple slope analyses for the late trauma, single trauma and non-interpersonal trauma groups (Fig. 2). In each of these groups, depression significantly predicted suicidal ideation at medium and high levels of avoidance (late trauma: medium, *B* = 0.05, *P* < 0.001; high, *B* = 0.08, *P* < 0.001; single trauma: medium, *B* = 0.05, *P* < 0.001; high, *B* = 0.08, *P* < 0.001; non-interpersonal trauma: medium, *B* = 0.03, *P* = 0.036; high, *B* = 0.07, *P* = 0.008).

## Discussion

The present study investigated the influence of depression and PTSD symptoms on suicidal ideation following exposure to traumatic events. Depression, PTSD symptoms and suicidal ideation were all found to be interrelated. Among the three PTSD symptom clusters, only avoidance demonstrated a significant moderating effect on the relationship between depression and suicidal ideation. Specifically, higher levels of avoidance strengthened the association between depressive symptoms and suicidal ideation. This moderating effect of avoidance was evident only among individuals exposed to late-onset, single-incident, non-interpersonal trauma; it was not observed in those exposed to early-life, repeated or interpersonal trauma.

Consistent with previous findings,^10–12^ PTSD symptoms were significantly associated with both depression and suicidal ideation. Moreover, the current study confirmed the association reported in prior research of a link between depression and suicidal ideation among trauma-exposed individuals.^8^

Partially supporting our hypothesis, only avoidance – among the three PTSD symptom clusters – moderated the relationship between depression and suicidal ideation. This effect was observed only when avoidance levels were moderate to high; under these conditions, depression was more strongly associated with suicidal ideation. These findings suggest that individuals who engage in increased levels of avoidant behaviours in response to trauma-related cues may face increased risk of suicidal ideation when experiencing depressive symptoms. Conversely, those with lower levels of avoidance may not exhibit heightened suicidal ideation despite being depressed following trauma exposure.

Unlike intrusion and hyperarousal, which are typically automatic responses, avoidance is a more intentional and effortful strategy^35,36^ that is often adopted as a coping mechanism to reduce psychological distress by evading trauma-related stimuli.^37^ However, excessive and prolonged reliance on avoidance may interfere with the extinction of conditioned fear responses, thereby intensifying negative emotional states and contributing to emotion dysregulation.^38,39^ Given that emotion dysregulation is a key mechanism linking depression to suicide,^40^ and that suicidal ideation can serve as an escape from such dysregulated affective states,^41^ greater avoidance may intensify the depression–suicidal ideation relationship through its deleterious effects on emotion regulation.

The moderating effect of avoidance was evident only in participants with specific trauma types, namely late-onset, single-incident and non-interpersonal trauma. By contrast, among individuals with early-life, repeated or interpersonal trauma, depression was associated with suicidal ideation regardless of avoidance level. When individuals are repeatedly exposed to trauma-related stimuli in the absence of adverse outcomes, they may experience reductions in anxiety and develop corrective beliefs.^42^ However, for those exposed to repeated trauma beginning in early life, confronting trauma-related cues may not have resulted in safety but rather reinforced the perception that such cues can still lead to negative outcomes. Through these early and recurrent experiences, maladaptive schemas may develop, characterised by the belief that trauma-related stimuli must be avoided to prevent retraumatisation. These schemas can disrupt information processing, impair adaptive responses to life events, and contribute to enduring psychological distress and dysfunction.^43–45^

Recovery following interpersonal trauma may require individuals to overcome avoidance of people and social contexts to experience safety through repeated, non-threatening interpersonal contact. However, interpersonal trauma can fundamentally alter interpersonal schemas related to safety, trust, control and intimacy, thereby undermining interpersonal functioning.^46^ As a result of these pervasive and negative beliefs about relationships, individuals with histories of interpersonal trauma may continue to feel unsafe even in objectively benign interpersonal situations. In other words, even when avoidance is reduced, they may maintain the belief that negative outcomes are inevitable. Furthermore, compared with non-interpersonal trauma, interpersonal trauma is more likely to motivate avoidance as a coping strategy, because it is accompanied by intense negative emotional states and may inhibit the development of the biological and psychological components of emotion regulation.^47^ This suggests that individuals with interpersonal trauma may adopt avoidance in a relatively rigid and trait-like manner, which may explain its non-significant moderating effect on the relationship between depression and suicidal ideation among the interpersonal trauma group. In addition, interpersonal trauma is often chronic, cumulative and complex in nature,^48^ which may account for the similar patterns observed in individuals with early-life, multiple and interpersonal trauma exposure.

Although therapeutic approaches to mitigating suicidal ideation in the context of PTSD and depression range from conventional clinical interventions to broader nature-based strategies,^49,50^ the observed importance of avoidance in suicidal ideation risk underscores the potential value of exposure-based interventions following trauma. Exposure therapy, a well-established treatment for PTSD, helps to correct maladaptive beliefs that anxiety and threat will persist unless trauma-related cues are avoided, thereby reducing both negative affect and avoidance behaviours.^51^ Such interventions may be particularly beneficial for individuals who have experienced acute, single-event or non-interpersonal trauma. In these cases, early implementation of exposure-based therapy could interrupt the progression from depression to suicidal ideation. Conversely, for those exposed to chronic or interpersonal trauma, exposure therapy may have limited efficacy. For these individuals, interventions that focus on preventing further trauma and targeting emotion dysregulation and maladaptive cognitive schemas may yield more favourable outcomes. Therefore, tailoring clinical interventions according to trauma type is essential to optimise therapeutic efficacy.

In addition, our findings suggest that assessment of both depressive symptoms and PTSD symptoms is important for identifying the risk of suicidal ideation among psychiatric out-patients with trauma histories. High levels of avoidance symptoms accompanied by depression should be considered to be a potential indicator of increased suicidal ideation. Furthermore, examining specific types of traumatic events during suicide risk assessment or screening could help to identify at-risk populations.

Several limitations should be considered when interpreting the findings of this study. First, participants were recruited from a single hospital, which may limit the generalisability of the results. Future studies should aim to include larger and more diverse samples drawn from multiple institutions. Second, owing to the cross-sectional design, no causal relationships among depression, PTSD symptoms and suicidal ideation could be established. In addition, cross-sectional moderation effects can be overestimated owing to common method variance, that is, spurious correlation created by using the same method to measure each variable.^52^ Longitudinal research is warranted to further examine the interactive effects of depression and PTSD symptoms on suicidal ideation over time. Third, as all participants were psychiatric out-patients, potential effects of comorbid psychiatric disorders and pharmacological or psychotherapeutic treatment on depressive severity and suicidal ideation cannot be excluded. Moreover, the findings may not be generalisable to individuals with trauma exposure but minimal psychiatric symptoms. Fourth, whereas trauma types were dichotomised in this study, future research should adopt more detailed classifications of trauma, including factors such as the number of traumatic exposures and the specific nature of the trauma experienced. Last, this study examined three core PTSD symptom clusters based on the DSM-Ⅳ framework. However, recent models propose an expanded symptom structure – including intrusion, avoidance, negative affect, anhedonia, externalising behaviours, anxious arousal and dysphoric arousal – which may provide a more comprehensive understanding of PTSD symptomatology consistent with DSM-5 criteria.^53,54^

This study demonstrated that avoidance moderates the relationship between depressive symptoms and suicidal ideation following trauma exposure. Specifically, at higher levels of avoidance, depressive symptoms were more strongly associated with suicidal ideation. This moderating effect was observed among individuals with late-onset, single-incident or non-interpersonal trauma, but not among those with early-life, repeated or interpersonal trauma. These findings highlight the potential value of interventions aimed at reducing avoidant behaviour for suicide prevention, particularly in individuals exposed to acute or non-interpersonal trauma.

## Data Availability

Data generated and/or analysed during the current study are available from the corresponding author upon reasonable request.
